# Surface/Interface Engineering for Constructing Advanced Nanostructured Photodetectors with Improved Performance: A Brief Review

**DOI:** 10.3390/nano10020362

**Published:** 2020-02-19

**Authors:** Meng Ding, Zhen Guo, Xuehang Chen, Xiaoran Ma, Lianqun Zhou

**Affiliations:** 1 School of Physics and Technology, University of Jinan, 336 Nanxinzhuang West Road, Jinan 250022, China; chenxuehang666@163.com (X.C.); mammxxxr@163.com (X.M.); 2 Key Lab of Bio-Medical Diagnostics, Suzhou Institute of Biomedical Engineering and Technology, Chinese Academy of Sciences, Suzhou 215163, China; 3 Zhongke Mass Spectrometry (Tianjin) Medical Technology Co., Ltd., Tianjin 300399, China; 4 Jihua Institute of Biomedical Engineering Technology, Jihua Laboratory, Foshan 528251, China

**Keywords:** photodetectors, interface/interface engineering, nanostructures, charge carriers

## Abstract

Semiconductor-based photodetectors (PDs) convert light signals into electrical signals via a photon–matter interaction process, which involves surface/interface carrier generation, separation, and transportation of the photo-induced charge media in the active media, as well as the extraction of these charge carriers to external circuits of the constructed nanostructured photodetector devices. Because of the specific electronic and optoelectronic properties in the low-dimensional devices built with nanomaterial, surface/interface engineering is broadly studied with widespread research on constructing advanced devices with excellent performance. However, there still exist some challenges for the researchers to explore corresponding mechanisms in depth, and the detection sensitivity, response speed, spectral selectivity, signal-to-noise ratio, and stability are much more important factors to judge the performance of PDs. Hence, researchers have proposed several strategies, including modification of light absorption, design of novel PD heterostructures, construction of specific geometries, and adoption of specific electrode configurations to modulate the charge-carrier behaviors and improve the photoelectric performance of related PDs. Here, in this brief review, we would like to introduce and summarize the latest research on enhancing the photoelectric performance of PDs based on the designed structures by considering their surface/interface engineering and how to obtain advanced nanostructured photo-detectors with improved performance, which could be applied to design and fabricate novel low-dimensional PDs with ideal properties in the near future.

## 1. Introduction

In recent years, following the developmental step forward of microelectronics technology, optoelectronic technology, as one of burgeoning advanced technologies, has developed rapidly and will have far-reaching effects on the lives of human beings. It involves optical display, optical storage, lasers, and other fields and is the core technology of the future information industry. It also has significant strategic importance for the state economy, technology, and defense. Various materials with different structures have attracted much more attention for their potential applications in integrated nano- or micro-optoelectronics, including light emitting diodes (LEDs) [1], solar cells [2,3], sensors [4,5], field-effect transistors (FETs) [6,7], photodetectors [8,9,10], and so on.

At present, smart photoelectric devices with excellent performance play important roles in our daily life, scientific research frontiers, high school education, and biomedical fields, and so on. The photodetector is actually a device that converts light signals into electrical signals. Compared with the classical photodetector devices with bulk materials, improved photoelectric conversion performance could be realized in constructed nanostructured photodetectors though from light absorption, photon charge carrier generation, and collection of contributing electronic signals, the field of which researchers have paid more and more attention.

Specially, a photodetector is an indispensable and important device in photoelectronic systems for military detection, aerospace satellites, and so on and helps scouts or researchers to collect key information for formulating strategies, which can also be widely used in national safety fields including flame monitoring and personal property safety [11], and so on. For an ideal photodetector, the main characteristics could be high sensitivity, fast response time, wavelength selectivity, and so on. Photodetectors could be classified in several ways: wavelength spectra (IR to UV detectors), information sampling (smoke, temperature, gas), structure (p–n junction; PN), metal-semiconductors (MS), and metal-semiconductor-metal (MSM). Photodetectors can be classified according to the dimension of the photodetector (PD): zero-dimensional (0D), one-dimensional (1D, such as nanowire, nanorod, nanotube, and so on), two-dimensional (2D, such as graphene (Gr) and transition metal dichalcogenides (TMDCs), three-dimensional (3D), or bulk. Semiconductor PDs have the unique advantages of high quantum efficiency, small size, low consumption energy, and high stability; therefore, PDs have attracted wide attention and interest of researchers.

So far, PDs based on single crystal, film, and nanostructured materials have been applied for building designed PDs to detect ultraviolet [12,13,14], visible [15,16,17,18], or infrared [19,20,21,22,23,24,25] photons with actual needs. For example, highly narrow band (bandwidth of 10 nm) solar-blind photodetectors of β-Ga_2_O_3_ single crystals with a peak responsivity of 0.23 A/W at 262 nm and an EQE of 110% were reported [26]. Metal-oxide-semiconductor ultraviolet PDs based on Au/MgO/MgZnO with high internal gain have been constructed [14]. A novel hybrid visible PD was realized using a planar p-type inorganic NiO layer in a junction with an organic electron acceptor layer. A hexagonal boron nitride (hBN)/b-As_0.83_P_0.17_/hBN sandwiched structured mid-infrared photodetector was constructed with responsivity of 190, 16, and 1.2 mA/W at 3.4, 5.0, and 7.7 μm at room temperature, respectively [20]. Conventional silicon (Si)-based PDs usually show peak photoresponse between 700 and 900 nm with rather low dark current because of the high crystalline quality and excellent passivation properties of Si [27]. Moreover, much research on flexible/stretchable image sensors based on thin-film Si PDs has been studied in materials science and engineering [28,29,30,31]. Especially, thin film silicon devices with nanophotonic structures could effectively improve the absorption efficiency of incident light [32,33], which was one of the directions to improve the performance of PDs. From the conclusion of the literature review, it could be illustrated that compared with film and bulk materials, low-dimensional nanostructures have the advantages of unique conductivity caused by high quality of crystal and carrier mobility and a confined carrier transport channel; thus, they are much more suitable candidate materials in assembling high-performance PDs on a large scale [34]. It could also be found that at this stage, various nanostructures of different materials have been utilized to fabricate PDs, including quantum dots (QDs) [24], nanoparticles (NPs) [35,36], nanowires [37,38,39], nanotubes [40], nanosheets [41,42], nanoribbons [43], nanobelts [44], and so on.

From the point view of dimension and easy assembly, nanowires are one of the best choices to be applied for building different kinds of photoelectric devices. The large surface-to-volume ratio and the presence of deep level surface trap states in NWs greatly prolong the photocarrier lifetime; the reduced dimensionality of the active area in NW devices shortens the carrier transit time. Indeed, the combination of long lifetime and short transit time of charge carriers can result in substantial photoconductive gain [45]. Especially, quasi-one-dimensional nanowires with semiconducting properties have been widely investigated as active materials for high-performance photodetectors [23]. It could be also explored from the published literature related to PDs that, generation, separation, transportation, and collection of photo-induced charge carrier are the key parameters for improving performance of the designed devices [12].

In general, the photoelectric conversion process from optical signals to electric signals in PDs mainly involves three steps:

(1) Generation of the photo-induced carriers in the case of external light radiation;
(2) Separation, transportation, and multiplication of photo-induced charge carriers derived by the applied electric field or built-in electric field formed at the interface of the heterojunction;
(3) Collection of the photocurrent generated by photo-excited carriers at both ends of electrodes, thus realizing the detection of external light radiation.

It can be observed that charge carrier generation, diffusion, and recombination modulation are all very important considerations in the construction of high-efficiency PDs [46]. The sensitivity, response speed, spectral selectivity, signal-to-noise ratio, and stability of detection are much more important factors to judge the performance of PDs [34].

To rationally implement surface and interface engineering in the PDs’ design, an understanding of the effects of surface and interface on reactions is required. In this section, we highlight a few of the key factors that need to be taken into account when designing nanostructures and their hybrid nanostructures. The carrier behaviors such as generation, recombination, separation, and collection are closely related to the surface/interface of devices, especially for the nanostructured devices; thus, surface/interface engineering of nanostructured materials is critically important for influencing the performance of the fabricated devices, and how to suitably apply surface/interface properties of nanostructured PDs for building advanced devices needs continuous exploration. In this review, how the surface/interface engineering improves the performance of the constructed nanostructured PDs is demonstrated theoretically through a review of the literature.

## 2. Surface and Interface States

**Figure 1:**
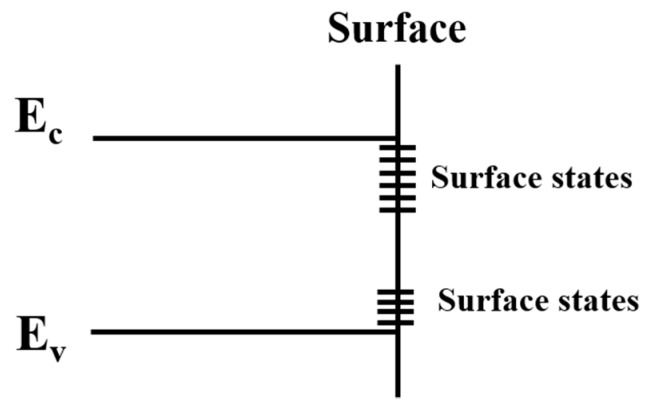
Schematic diagram of surface level.

A surface could be defined as atomic layers that do not have three-dimensional continuous environment of bulk materials, three-dimensional continuous environment, or the periodicity of the infinite lattice that is destroyed by the existence of nanostructured surface. For crystal structures, in the direction of the vertical surface, the potential energy of lattice atoms could not have corresponding symmetry, and some new eigenvalues could be obtained in the Hamiltonian characteristic value when the Schrodinger equation is applied through the theory of quantum mechanics [47]. A new energy level could appear and be defined as a surface state, as shown in Figure 1. There are actually two kinds of surface states: intrinsic and external surface states. The intrinsic one is the surface state without foreign impurities, and the other is due to the existent of impurities from adsorbed atoms or other imperfections on the surface.

Semiconductor surface research is mainly concerned with various electrical properties of surface phenomena. These phenomena involve the free carriers in the space charge region, surface states, and their mutual interactions. Although the free carrier transport in the space charge layer should be paid considerable attention, the most concerted effort in the study of the surface electrical properties has been directed towards the surface states [48]. Much research on semiconductor surfaces caused by cleaving in ultrahigh vacuum suggested that the formation of surface states is attributed to dangling bonds at the surface [49]. According to a previous report [47], the schematic diagram of the surface level is shown in Figure 1. The surface states could also be divided into donor states or acceptor states based on the behavior of the electrons. Surface states could capture, scatter generated carriers, and influence electric field effect, and the results could be effective photon capture, carrier generation, and combination centers, which controls surface properties of the absorbed photons and generated carriers. Scattered carriers influence surface mobility, thus affecting surface conductance.

An interface could be defined as the interface between heterostructure phases, similar to surface states. Interface states that are introduced at the interface state occur at the interface where two heterostructure media exist and contact. Thus, the continuous environment of atoms or lattice structures is destroyed by the existence of other substances. Hence, the interface states could be introduced by other atoms, lattice mismatch, interface roughness, and thermal expansion of two materials’ interface. Generally speaking, there are two interface states: donor and recipient. Considering energy levels of the built heterostructures, the existing interface energy levels greatly influence carrier transport behaviors, which controls performance of the devices. The performance of the photodetector devices could be considerably improved by considering interface carrier transport properties by designing optimal heterostructures.

Commonly, interface engineering includes surface charge transfer, charge injection, and collection on the metal electrode/semiconductor interface and carrier bound state on the dielectric/semiconductor interface. On the other hand, there are a large number of defects in lattice structure, including vacancies, adsorbed atoms, grain boundaries, and impurities. Interfaces and defects have a critical influence on the properties and operational stability of metal halide perovskite optoelectronic devices. Therefore, interface and defect engineering is crucial to control the behavior of the charge carriers and to grow high quality, defect-free perovskite crystals. Yang et al. summarized the strategies of interface and defect engineering in perovskite solar cells and light-emitting diodes [50]. Due to the atomic thickness and super high specific surface area of two-dimensional TMDCs, the interface of TMDCs plays a decisive role in the device performance. Chen and other researchers summarized and highlighted advances in TMDC interfaces and defect engineering and applications in electronic devices. Various appropriate interfaces and defect engineering that effectively adjust the electrical and optical properties of the TMDCs were presented in order to achieve the ultimate goal of improving device performance [51]. By adopting appropriate defect engineering strategy, the defect fix, controlling the type and concentration of carriers, and the reduction of contact resistance could be achieved to realize high-performance electronic devices. On the other hand, controlling the defect state could increase the sensitivity of the photoconductive device or improve the response speed of the device. A heterojunction can be constructed to produce a photovoltaic effect through defect doping [52], and also the quantum electroluminescence effect can be produced using defects reasonably [53].

## 3. Surface/Interface Engineering for Improvement of Photodetector Properties

For the design and fabrication of optoelectronic devices based on nanostructures, the surface-to-volume ratios, interface areas, and interaction between surface and environment are increased dramatically, which has great impact on the performance of a PD device. For example, trapping at surface states of ZnO nanowire obviously influenced the transport and photoconduction properties of nanowires. The high surface-to-volume ratios and the existence of deep level surface trap states in nanostructures greatly extend the lifetime of photogenerated carriers. Moreover, the low dimensionality of the active area in a nanostructure device can reduce the transit time of carriers [45]. Zhou and colleagues fabricated UV photodetectors based on single In_2_O_3_ nanowire, and the conductance was significantly increased by four orders of magnitude [54]. Fang et al. constructed individual SnO_2_ nanowire UV photodetectors, which showed excellent optical selectivity and ultrahigh external quantum efficiency [55]. Yang et al. reported the ZnO nanowire UV PD with high internal gain, which was attributed to the presence of oxygen-related hole-trap states at the surface of the nanowire. At the same time, the slow dynamics of the surface oxygen molecules’ adsorption and desorption processes could result in a long response time in both the rise and decay process of a PD [45,56]. That is, the major shortcoming for ZnO based on PD applications is the strong persistent photo-induced conductivity after light illumination, which inhibits a fast recovery of the dark current [57]. Moreover, the persistent photoconductivity phenomenon was observed in PDs based on MoS_2_ [58,59]. The performance of PD devices can be improved by the following ways: (a) enhancing the interaction between light and matter; (b) decreasing the adverse effects of defects; and (c) adjustment of electronic characteristics [60]. Therefore, various approaches, such as doping [61], surface functionalization [56,62,63,64,65,66,67,68], surface carrier transport modulation [34], interface carrier-trapping/transport control [46], piezo-phototronic effects [69,70,71,72,73,74], and so on, have been utilized to improve the photoresponse performance of low-dimension nanostructure PDs for use in practical applications. The following section lists several ways to deal with surface/interface issues for improving performance of the photodetectors.

### 3.1. Surface-State Passivation for Terminating Dangling Bonds

**Figure 2:**
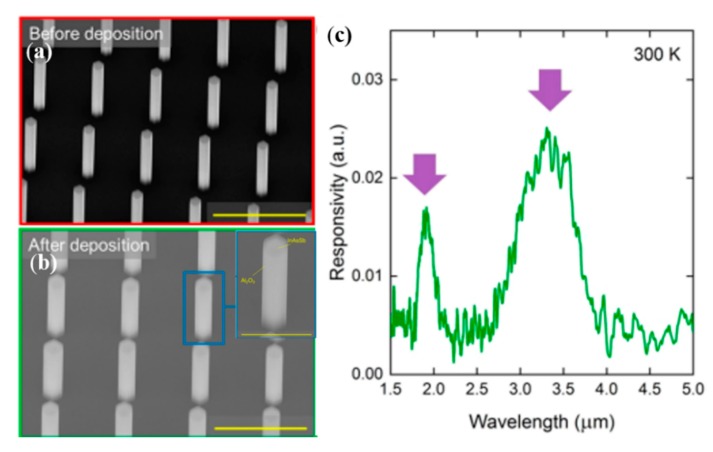
SEM images of (**a**) InAsSb nanowires and (**b**) InAsSb nanowires with ∼60 nm Al_2_O_3_ passivation, the scale bar is 2 μm. The inset is single nanowire coated with Al_2_O_3_ passivation layer, the scale bar is 1 μm. (**c**) Spectral response at a reverse bias of 0.5 V at room temperature [38].

As nanostructured photodetector devices have a much larger surface to volume ratio, and surface states play an important role in controlling their performance, surface-state passivation was one of the most useful solutions to deal with the mentioned issues. Surface-state passivation has been considered as one of the most effective and advanced methods to improve the performance of PDs through terminating their dangling bonds to decrease their surface states’ influences. Fang et al. [64] modified the ZnO nanowalls by CdS nanoparticles and investigated the effect of CdS nanoparticles on the optical and photoelectrical properties of a PD. The surface states of ZnO nanowalls was suppressed due to the introduction of CdS nanoparticles’ passivation layer; thus, the deep-level emission was reduced, and the recombination of carriers was prevented, and then the photoconductivity of ZnO nanowalls was improved obviously. Ren et al. [38] constructed nanowire-based photodetectors at mid-wavelength infrared composed of vertical selective-area n-InAsSb nanowire photoabsorber arrays on large bandgap p-InP substrate. In order to effectively inhibit the nonradiative recombination at the surface of InAsSb nanowire, the Al_2_O_3_ passivation shells were introduced, as displayed in Figure 2a,b. Furthermore, it was demonstrated that the photoluminescence (PL) emission intensity of InAsSb nanowire arrays with Al_2_O_3_ passivation layer increased 10- to 50-fold at 77 K. The spectral response of n-InAsSb/p-InP PDs with Al_2_O_3_ passivation at a reverse bias of 0.5 V is shown in Figure 2c. Two detection peaks located at about 2.0 and 3.4μm were observed at room temperature.

**Figure 3:**
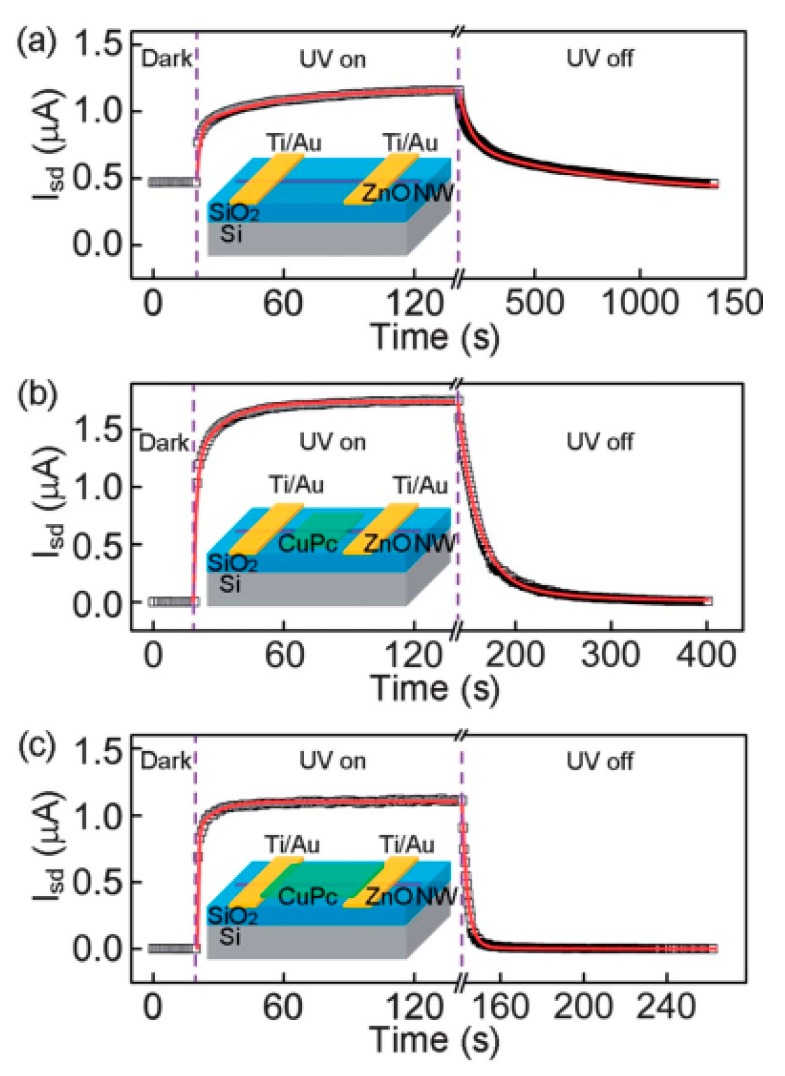
(**a**) The photoresponse curve of the bare ZnO nanowire device, (**b**) the partial coverage device, (**c**) the full coverage device. The insets are schematic diagrams of corresponding devices, respectively. The photoresponses of three devices with different CuPc coverage were measured at bias voltage of 0.5 V, and the UV illumination source was 350 nm light with intensity of 100 uW·cm^−2^. [56].

Yang et al. reported the single ZnO nanowire PDs with large photoresponse and high internal gain; the hole-trapping mechanism was also proposed. The holes could be trapped through oxygen adsorption and desorption at surface states caused by the dangling bonds, further prolonging the lifetime of photogenerated carriers, and multiple electrons could transit through the nanowire. Finally, the photoconductive gain was realized [45]. However, the surface adsorption process could make the response time (rise and decay) of PDs become large, which is unfavorable for the performance of PDs. Chen et al. [56] constructed photoconductive ZnO nanowire/copper phthalocyanine (CuPc) hybrid PDs. A p–n heterojunction was formed at the interface of CuPc film and ZnO nanowires, leading to reduced conductivity and inhibited dark current in the ZnO NW. Under UV illumination, electron–hole pairs were generated and separated by a built-in internal electric field, and photoinduced potential was built. Meanwhile, the width of the surface depletion layer reduced, resulting in higher conductivity and increased photocurrent. The transition was rather fast and did not consider the adsorption or desorption process of molecules; thus, the rise and decay time of ZnO nanowire/CuPc photoresponse was shorter than that of the ZnO nanowire, which is shown in Figure 3. Therefore, the main reason for the improved photoresponse speed was the passivation of ZnO nanowires’ surface states by CuPc film.

**Figure 4:**
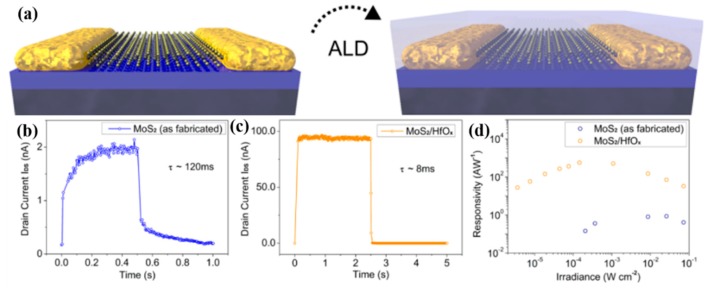
(**a**) Device schematic of MoS_2_ and MoS_2_/HfO_2_ photodetectors (PDs). The photocurrent of MoS_2_ before (**b**) and after (**c**) HfO_2_ encapsulation. (**d**) The power-dependent responsivity of a bilayer MoS_2_ device before and after HfO_2_ encapsulation [82].

For two-dimensional nanomaterials, many reports about transition metal dichalcogenides (TMDCs) with layer structure material and interacting layers held together with weak van der Waals interaction, including MoS_2_ [75], MoSe_2_ [76], WSe_2_ [77], WS_2_ [78], and ReS_2_ [79], have been published. Because of their large surface volume ratio, the performance of these detectors is more sensitive to the environment. Studies have shown that physically adsorbed gas molecules, such as O_2_ and H_2_O, could deplete MoS_2_ and MoSe_2_ by removing electrons from the channels [80,81]. Konstantatos et al. constructed a high-stability and excellent-performance photodetector with an atomic layer deposited hafnium oxide (HfO_2_) encapsulated monolayer and bilayer MoS_2_, and the schematic of the PD device is shown in Figure 4a [82]. The oxide could suppress strong current drifting and degradation caused by environmental effects and effectively remove the surface atmospheric adsorbates. Unprotected MoS_2_ photodetector devices usually show slow response speed, which is displayed in Figure 4b. After encapsulation with HfO_2_, the photocurrent of the PD device was increased by about 40 times, while the decay time was reduced by more than one order of magnitude, as shown in Figure 4c. Moreover, the responsivity of MoS_2_ PDs was promoted by more than one order of magnitude after HfO_2_ encapsulation, as shown in Figure 4d. Therefore, the investigation of effective packaging technology and the prior complete removal of surface binding adsorbates is an effective way to achieve high speed and sensitive light detection.

Certainly, other research groups have also used surface passivation to improve the performance of different detectors. Ni et al. investigated the influence of the MgO surface modification on the photocurrent and responsivity performance of TiO_2_@MgO core-shell nanowire array self-powered UV PDs [66]. The energy level structure between TiO_2_ and MgO is beneficial to the process of the separation of photogenerated electron–hole pairs and thus restrains the recombination of photogenerated carriers. Chen’s group [61] have utilized a surface transfer doping method to adjust the electronic properties and work function of graphene films and then tune the Schottky barrier of Gr/Si junctions. The transition metal oxide molybdenum trioxide (MoO_3_) possesses high work function [83] and has displayed obvious surface transfer hole doping effects in graphene, MoS_2_, and other two-dimensional materials [67]. Therefore, they fabricated a Gr/Si self-powered photodetector using in situ surface functionalization of the MoO_3_ layer on graphene. It was demonstrated that the photocurrent responsivity of MoO_3_ doped Gr/Si PDs was significantly improved for a wide spectrum ranging from ultraviolet to near infrared. In addition, the external quantum efficiency, photocurrent responsivity, photovoltage responsivity, and the specific detectivity (D*) of Gr/Si devices were significantly promoted after MoO_3_ decoration, which was attributed to the more efficient photocarrier separation and collection process caused by the increased height of the Schottky barrier at the Gr/Si interface and the reduced series resistance of the Gr/Si device after MoO_3_ modification [61]. Sangyeon Pak [84] demonstrated the effects of surface functionalization on charge carrier density and photoresponse performance of the MoS_2_ photodetector.

### 3.2. Surface Plasmonic Resonance for Strong Scattering and Absorption of Incident Light

**Figure 5:**
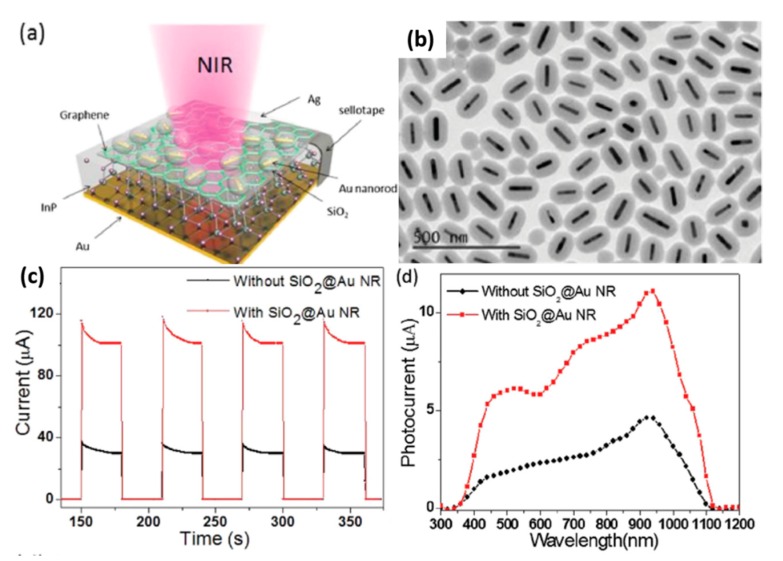
(**a**) The schematic diagram of the SiO_2_@AuNRs-SLG/InP PD device. (**b**) The TEM image of the SiO_2_@AuNRs on single layer graphene film. (**c**) Photoresponse of two different devices with and without SiO_2_@AuNRs modification under 980 nm light illumination of 6.7 mW cm^−2^ without applied bias. (**d**) Photoresponsivity of the NIR PDs with and without SiO_2_@Au decoration [96].

In recent years, many research groups have found that metal-surface plasma could be used to enhance the performance of detectors, which was attributed to the localized surface plasmon resonance (LSPR) effect, resulting in strong scattering and absorption of incident light, effectively separating photocarriers and transporting carriers at the interfaces of metal/semiconductor. Metal surface plasma is a hybrid electromagnetic wave mode formed by coupling of free vibrating electrons and incident photons on the metal surface. Both conductive surface plasmas at metal/dielectric interfaces and localized surface plasmas on metal nanoparticles have unique optical properties: high spatial local properties and local field enhancement properties. The unique photonic properties of surface plasmas make them widely used in subwavelength sensors, detectors, and modulators. To date, various plasmonic structures have been explored to improve the performance of photodetectors or phototransistors. In general, noble metals, for example Au [85,86,87,88,89], Ag [90,91,92], and Pt [93,94,95], are used as plasmonic metals for their useful range in the visible wavelengths, due to the excellent optical property as a result of SPR. In the development of plasmonic photodetectors using Au@MoS_2_ heterostructures, the photoresponsivity of the Au@ MoS_2_ device was about 10 times larger than that of planar MoS_2_ devices. Another type of Si-supported Au@MoS_2_ p−n junction photodiode demonstrated photoresponsivity of 10−30 A/W, higher than the values reported for similar MoS_2_ gateless photodevices, and the response time was less than 20 ms [88]. Luo et al. demonstrated that the photodetector device modifying plasmonic Au nanostructures onto the surface of CdTe NW exhibited the obvious photoresponse to 510 nm light illumination, with high response speed and fast recovery time [87]. A high-performance near-infrared (NIR) PD was constructed by coating single layer graphene (SLG)/InP Schottky junction diode with plasmonic SiO_2_@AuNRs; the schematic diagram of the PD and the TEM image of the SiO_2_@AuNRs on SLG film are shown in Figure 5a,b [96]. The decoration of SiO_2_@AuNRs could slightly improve the Schottky junction barrier, resulting in the increased built-in electric field, and then the separation efficiency of carriers was improved. The light trapping effect of the SiO_2_@AuNRs-SLG/InP device nearly promoted the absorption of incident light; as a result, many more holes and electrons were generated, and therefore photocurrent and responsivity were increased obviously (displayed in Figure 5c,d). Furthermore, the response rate of the SiO_2_@AuNRs-SLG/InP device was rather fast, which was able to monitor switching optical signals with a frequency up to 1 MHz, indicating its potential application in sensing high-frequency optical signals.

It was demonstrated that by coating Ag metal nanoparticles onto ZnO nanowires, the UV photodetectors exhibited higher sensitivity by four orders of magnitude with rather fast and stable response speed [90]. Arquer and colleagues reported that by introducing Ag metal nanoparticles into the PbS colloidal quantum dot photoconductive photodetectors, the absorption ability was increased due to a plasmonic scattering layer of Ag metal nanoparticles. As a result, the responsivity enhanced about 2.4-fold in the near infrared with the absorption band edge of ~1 μm [91]. Apart from noble metals, aluminum (Al) has been reported as a viable plasmonic metal for its useful range in the UV wavelengths [97,98]. Unlike the noble metals, the localized plasmon resonances can extend from the visible spectrum to the ultraviolet because the d-band of Al lies above its Fermi energy [99]. The Al element is a kind of abundant and low-cost metal found in the world, which is a better plasmonic material than Au and Ag in the UV range due to the negative real part and relatively low imaginary part of its dielectric function [100]. Xu et al. [97] improved the response characteristic of ZnO nanorod array UV PDs using surface plasmonic resonance by Al nanoparticles. Compared with the pure ZnO nanorod array PDs, the responsivity of the ZnO nanorod photodetector modified with Al nanoparticles was improved from 0.12 A/W to 1.59 A/W.

**Figure 6:**
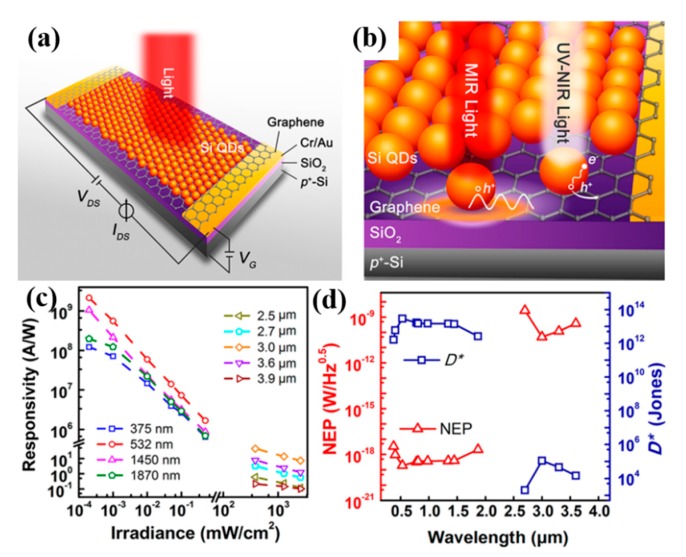
(**a**) Schematic diagram of B-doped Si-quantum dots (QDs)/graphene PDs. (**b**) Two different optical phenomena of B-doped Si QDs. (**c**) The irradiance power-dependent responsivity of the Si-QD/graphene PDs with different laser wavelengths at V_G_ = 0 V and V_DS_ = 1 V. (**d**) The spectral dependence of the noise equivalent power (NEP) and D* of the PDs. The measurements were performed at room temperature and 77 K, respectively [103].

Recently, it has been observed that boron (B)-doped and phosphorus (P)-doped silicon (Si) nanocrystals enable the localized surface plasmon resonance of Si nanocrystals in the mid-infrared (MIR) region [101]. Especially, the heavily B-doped silicon nanocrystals induced band-tail states that could expand the optical absorption of Si from the UV-visible region into the near-infrared region. Furthermore, the localized surface plasmon resonance of heavily B-doped silicon nanocrystals could be tunable, which is attributed to free holes above the Fermi level from the B-induced impurity band [102]. Yang et al. [103] constructed quantum dots (QDs)/graphene hybrid MIR PDs by using plasmonic B-doped Si QDs; the schematic diagram of B-doped Si-QDs/graphene PDs is illustrated in Figure 6a. In the process of operating PDs, two different optical phenomena of silicon quantum dots can be adopted, as shown in Figure 6b. In the MIR region, the LSPR of silicon quantum dots generated a strong electric field that increased the direct excitation of the underlying graphene. Therefore, the phototransistor could effectively respond to the MIR light. The UV-to-near-infrared absorption of B-doped Si QDs resulted in the generation of electrons and holes in quantum dots. The photoresponse of the PD device was obtained by transferring one type of carrier from the quantum dot to graphene and capturing another carrier in the quantum dot. Figure 6c displays that the responsivity of Si-QD/graphene PDs was reduced with the increase of the irradiance power. The responsivity was in the range of 1.2 × 10^8^ to 2.2 × 10^8^ A/W with the UV-to-NIR light illumination at the lowest irradiance of 0.2 μW/cm^2^. As can be seen from Figure 6d, the noise equivalent power (NEP) values of the PDs in the MIR region and the UV-to-NIR region were about 10^−10^ and 10^−18^ W/Hz^0.5^, respectively. The fairly small NEP value suggests that the Si-QD/graphene photodetector can be applied to low light detection. The values of D* were about 10^5^ and 10^13^ Jones for the MIR and the UV-to-NIR photodetection, respectively.

### 3.3. Interface Carrier-Trapping/Transport Modulation

Once the design principles are defined, important parameters of interface engineering can be directly identified, containing interface compositions, areas, defects, faces, electronic coupling, and band bending. Before the interface engineering, the roles of the interface in the design structure should be fully understood according to the specific charge dynamics models [104]. For example, defects at the interface tend to be the recombination centers of electrons and holes, thus hindering the charge transfer across the interfaces. Therefore, eliminating the interfacial defects can improve the performance of the PDs in most situations. It has been proved that improving the quality of crystals, optimizing the arrangement of energy levels of layers, and constructing p–n or Schottky heterojunctions could be effective interfacial engineering methods to improve the performance of photodetectors [105].

**Figure 7:**
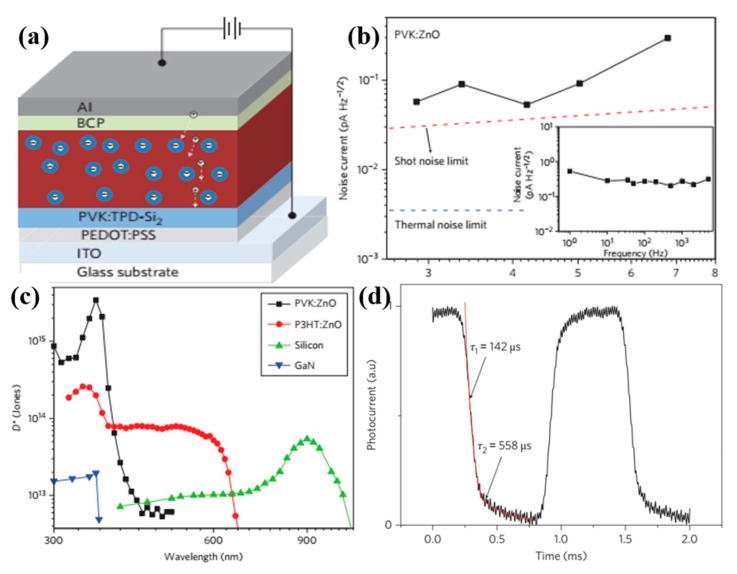
(**a**) The schematic diagram of the PD device. (**b**) Noise characteristics of the PVK/ZnO PDs under different currents, the inset is the frequency-dependent noise current with −9 V bias. (**c**) Specific detectivities of the photodetector at different wavelengths. (**d**) Transient photocurrent of the P_3_HT/ZnO device [106].

It is demonstrated that surface and interface engineering play a key role in improving the performances of PDs. The generation, diffusion, and recombination/transport processes of charge carriers can influence the performance of PDs; thus, these factors should be taken into account when the PDs are built. Huang et al. [106] have constructed a new type of nanocomposite ultraviolet photodetector; the schematic drawing of the PD is shown in Figure 7a. The Frenkel excitons generated in ZnO nanoparticles and polymers could diffuse to the polymer/nanoparticles interface. Under reverse bias voltage, holes were transported in the semiconducting polymer, but the electrons were still trapped in the nanoparticles, attributed to the short percolation network for electrons and the strong quantum confinement effect of ZnO nanoparticles. Because of the strong electron trapping effect, the PDs had Schottky contact in the dark and an ohmic contact under illumination, confirmed by the dark current and photocurrent. That is, the PD device transitioned from a photodiode (in dark) to photoconductor (under illumination) via interfacial trap-controlled charge injection. The measured total noise current in the frequency range 1 to 5 kHz was mainly the shot noise, as shown in Figure 7b. The specific detectivity of hybrid PDs (shown in Figure 7c) was tens- to hundreds-fold better than that of inorganic semiconductor photodetectors. Furthermore, the response speed of the PD was rather high compared to other photodetectors based on any nanoparticles or colloidal quantum dots. It was indicated that two channels for the recombination of holes existed through the decay of the photocurrent experiment (displayed in Figure 7d). The decay process was combined with a fast component of 142 ms and a slow component of 558 ms.

Semiconductor heterojunctions with different bandgaps could exhibit different interface properties. By adjusting the arrangement of interfacial energy bands, the heterojunction structures may possess unique photoresponse characteristics. Moreover, when constructing the heterojunction for multicolor photodetection, it is necessary to have a large band deviation at the interface of the heterojunction in order to effectively extract carriers. There are larger band offsets between the Si/TiO_2_ heterojunction; according to the calculation, the conduction band offset (Δ*E_C_*) and the valence band offset (Δ*E_V_*) were 0.75 eV and 2.66 eV, respectively, which would be conducive to control the transport of the carriers. Hu et al. [107] fabricated a multicolor photodetector based on the n-Si(111)/TiO_2_ nanorod array heterojunction ranging from ultraviolet to visible light by controlling the applied voltage. In the case of forward bias, the band offsets would not hinder the diffusion of carriers; thus, there was no difference between the photocurrent and the dark current. The PD device exhibited multicolor detection capability under reverse bias because the motion of these carriers was controlled by the applied bias and the barrier of conduction band offset and the valence band offset.

**Figure 8:**
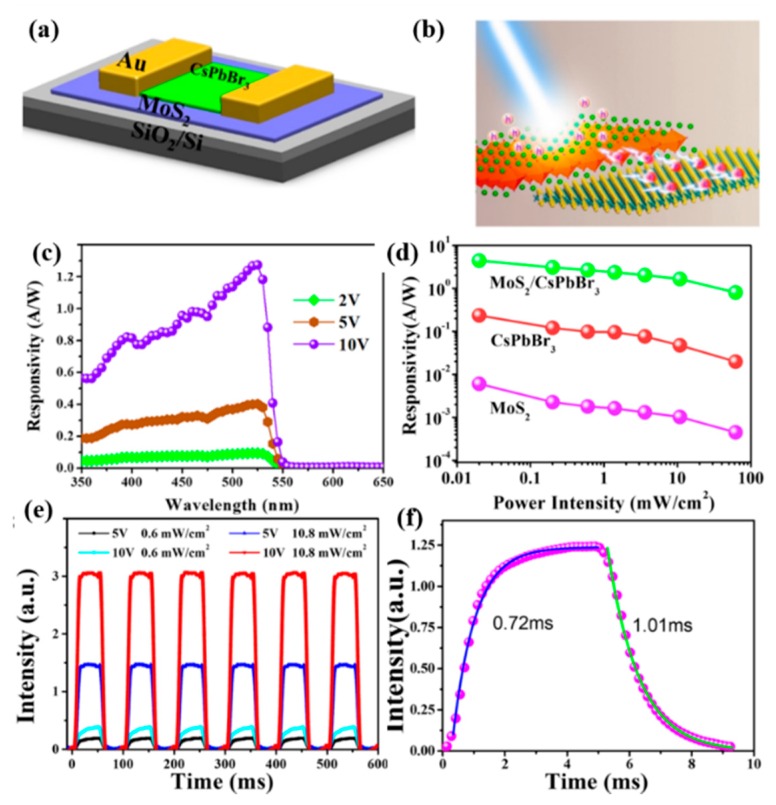
(**a**) The schematic diagram of the hybrid MoS_2_/CsPbBr_3_ PDs. (**b**) The schematic diagram of the charge transfer from CsPbBr_3_ to MoS_2_ under illumination. (**c**) Photoresponsivity spectra of the hybrid MoS_2_/CsPbBr_3_ PDs with different applied bias of 2, 5, and 10 V. (**d**) Photoresponsivity of the three PDs as a function of the power intensity. (**e**) Cyclic response of the hybrid MoS_2_/CsPbBr_3_ PDs at different voltages and incident optical power intensity under 442 nm laser illumination. (**f**) The temporal photocurrent response of the hybrid PDs with rising time (0.72 ms) and decay time (1.01 ms), respectively [108].

Nowadays, Perovskite material has become one of the superstar media for constructing different kinds of photoelectric devices; especially, all-inorganic cesium lead halides (CsPbX_3_, X = Cl, Br, I) represent an emerging class of materials owing to their high carrier mobility, long carrier diffusion length, and excellent visible light absorption. The high quantum efficiency (over 90%), narrow line width, and high stability make these all-inorganic perovskites suitable for application in novel optoelectronics [108]. Song et al. [108] utilized the advantages of large absorption coefficient and high quantum efficiency of the perovskites by fabricating the superior performance hybrid CsPbBr_3_ perovskite/MoS_2_ PD with a high photoresponsivity of 4.4 A/W and an external quantum efficiency of 302%; the schematic diagram of the hybrid PDs is displayed in Figure 8a,b. Especially, the photogenerated electrons in the perovskite were effectively transferred and injected to MoS_2_. MoS_2_ monolayer acted as an electron-collecting layer for perovskite photodetectors to improve the photoelectrical response, as shown in Figure 8c–e, which could decrease the probability of carrier recombination; therefore, the photoresponsivity of the hybrid CsPbBr_3_ perovskite/MoS_2_ PDs improved by three orders of magnitude compared with the pure MoS_2_ PD. The dark current was also obviously reduced. Furthermore, the MoS_2_ layer could induce the trap passivation on the substrate and benefit the carrier transport and then improve the separation efficiency of carriers; thus, the rising time of the hybrid PD was also lowered from 65.2 to 0.72 ms after combining with MoS_2_ layers, as displayed in Figure 8f. It was demonstrated that 2D nanomaterials combined with the perovskite layers could be considered as a superior candidate to realize the next-generation high-performance photodetectors.

**Figure 9:**
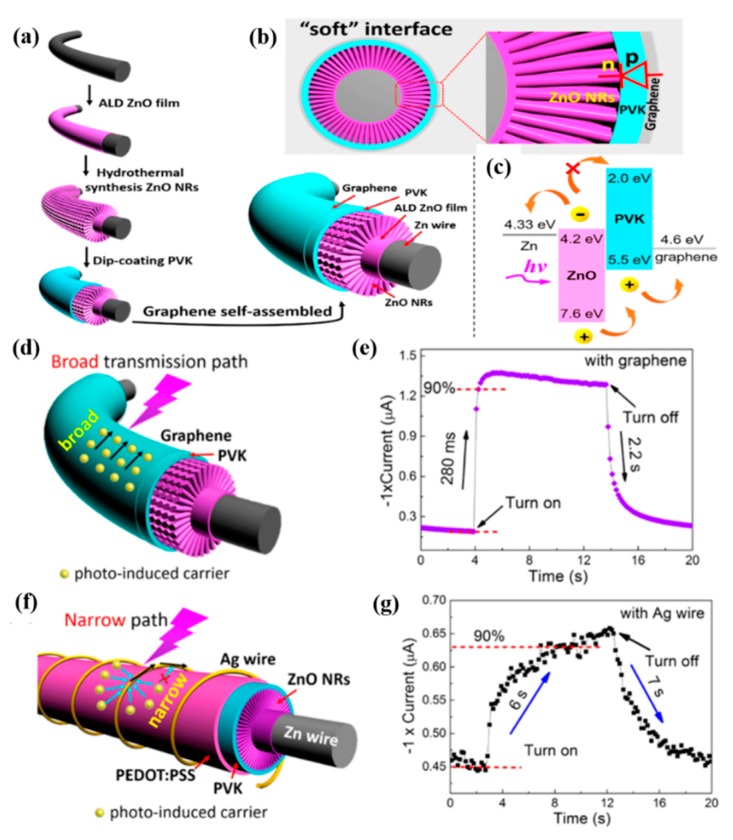
(**a**) Schematic diagram of the preparation process procedure of hybrid fiber-shaped photodetector based on ZnO NRs array/PVK/graphene. (**b**) The cross-sectional diagram of the device that revealed the p–n heterojunction and all tight soft interfaces in the PDs. (**c**) Schematic diagram of the corresponding energy levels of the material involved in the PD device, as well as the transportation of electrons and holes caused by the arrangement of interfacial energy levels. (**d**) Schematic representation of the transport property of the photoinduced carriers in the PD with structure of ZnO/PVK/graphene. Photoresponse characteristics of (**e**) corresponding current–time curves of ZnO/PVK/graphene PDs. (**f**) Schematic representation of the transport property of the photoinduced carriers in the PD with structure along Ag wire. (**g**) Corresponding current–time curves [105].

For fiber-shaped devices, the interface problems of the fiber device were more serious, which was attributed to the curved and rough surface, many more defects, and poor contacts. Hence, smoothing the rough surface and achieving better contact between all layers was particularly important for improving the performance of fiber-shaped devices. Zeng et al. [105] built and constructed an inorganic-organic-graphene hybrid fiber-shaped PD via “soft” interfaces of all layers; the schematic diagram illustrating the fabrication procedure of ZnO nanorods/PVK/graphene hybrid fiber-shaped PDs is displayed in Figure 9a. In this hybrid fiber-shaped PD, the organic semiconductor completely covered the inorganic functional layer, forming “soft” contact and smoothing its rough surface. In addition, the ultra-soft graphene could tightly wrap around the surface, creating another “soft” interface, as shown in Figure 9b. These “soft” interfaces between the various functional layers even at curved interfaces have established the tight contact; thus, the contact resistance could be significantly reduced. The ZnO nanorods array with good crystal quality was uniformly grown on the Zn wire. As seen in Figure 9d,e, the rise time of the PD with structure of ZnO/PVK/graphene was about 280 ms with fast response speed. Meanwhile, by using hard metal Ag wire instead of soft graphene as surface electrode, the response speed slowed down and the rise time of PDs increased to 6 s, as shown in Figure 9f,g. Therefore, the tight contact and reasonable interfacial energy level alignment (shown in Figure 9c) were beneficial to the separation and transportation of photogenerated electrons and holes. The results showed that the performance of the fiber-shaped PDs could be significantly improved by effective interface optimization.

**Figure 10:**
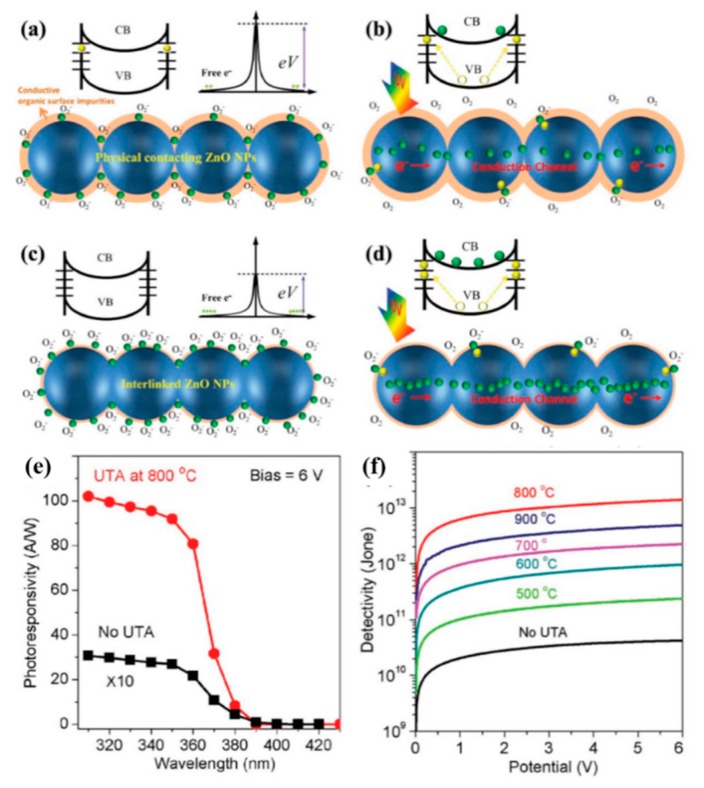
(**a**,**b**) The ZnO-NP before ultrafast thermal annealing (UTA). Schematic diagram of the energy band (top) and the photoresponse mechanism (bottom). (**c**,**d**) the nanojunction-interlinked ZnO-NP networks after the UTA treatment. The orange shell on ZnO-NPs represents the surface defects and contaminants. Green and yellow dots denote electrons and holes, respectively. (**e**) Spectral responsivity of the same PDs before and after UTA treatment at 800 °C for 2 s under the 340 nm UV illumination. (**f**) The relationship between applied bias and detectivity for a set of samples treated with UTA at different temperatures ranging from 500 to 900 °C [109].

Furthermore, interface nanojunction engineering of the electron depletion effect could be utilized to improve the performance of PDs. For instance, Liu et al. [109] constructed nanojunction-interlinked ZnO nanoparticle networks without changing the dimension and morphology of the nanoparticles using an ultrafast thermal annealing (UTA) method. Figure 10 indicates band bending, electric field, and the low conductivity surface depletion layer present due to the adsorption of oxygen molecules on the ZnO nanoparticle surfaces. Contaminants (orange shell in schematic) and surface defects could reduce the electron depletion effect, resulting in high dark current and low photocurrent, as shown in Figure 10a,b. Surface modification using UTA can eliminate surface defects and contaminants and thus restore the electron depletion effect. Finally, this caused a decrease in the dark current and an increase in photocurrent, as shown in Figure 10c,d. In addition, ZnO NP–NP interface nanojunctions could be fused into interlinked ZnO-NP networks, which could significantly reduce the potential barrier of inter-NP and thus suppress the recombination of carriers in the transport process. From Figure 10e it is evident that the photoresponse of PDs increased dramatically by about four orders of magnitude with the decreased wavelength across the band edge after UTA treatment at 800 °C for 2 s. Figure 10f displays the relationship between applied bias and detectivity for a set of samples treated with UTA at different temperatures in the range of 500 to 900 °C. The detectivity value increased at first and then descended with the increasing UTA temperature and obtained the maximum value of 1.4 × 10^13^ Jones for the sample treated at 800 °C. Especially, D* after UTA treatment at 800 °C was enhanced by approximately three orders of magnitude compared with that of untreated, demonstrating the significance of removing surface defects and contaminants and improving the interconnections of NP. Therefore, it is very important to achieve high-performance detection making full use of the electron depletion effect with the minimum charge combination.

### 3.4. Piezo-Phototronic Effects for Modulating Carrier Transport Behavior

In fact, piezoelectric phenomena exist in materials such as semiconductors, polymers, ceramics, and even biological media. Among them, the well-known piezoelectric materials are quartz and Pb(Zr, Ti)O_3_, but the non-semiconductor and insulating properties limit their applications in photonic and electronic fields [110,111]. In theory, any semiconductor material with non-central symmetry could produce piezoelectric effect. Generally speaking, ZnO with wurtzite structure is the most well-known material in the study of the piezoelectric effect; it not only has rich nanostructural morphology but also possesses much higher piezoelectric coefficients than other II–VI family materials. The transmission characteristics of carriers can be dynamically adjusted by using the effect of piezoelectric potential, so that they have potential application prospects in mechanical electronic devices, including sensors, nanorobots, and so on. When considering the interaction with light, by adjusting the magnitude of the strain and the intensity of light, the transmission properties of carriers in the device can be effectively modulated. Light-emitting diodes [112,113], solar cells [114,115,116], and photodetectors [74,117,118,119,120] associated with the piezo-phototronic effect have attracted wide attention because of their potential application prospects. The band structure and carrier transport behavior can be dynamically modulated by taking advantage of the piezo-phototronic effect in the photodetectors.

The piezo-phototronic effect could be applied in different ways with designed structure [111,114]. Wang et al. [121] utilized the piezo-phototronic effect to adjust the height of the Schottky barrier at local contact; the performances of the Schottky contact metal-semiconductor-metal photodetector based on the GaN nanobelt was enhanced. Generally, the response ability of the PDs was obviously improved due to the piezotronic effect when a strain was applied on devices. In particular, the responsivity of PDs increased by 18% with a compressive strain, and their sensitivity increased from 22% to 31% [121]. The piezo-phototronic effect can not only improve the responsivity of the PDs but can also adjust the dark current of the detector. Wang et al. [122] constructed MSM ZnO micro-/nanowire PDs; the responsivity of the PDs was increased by 530% under −0.36% compressive strain, while the dark current of PDs was tuned with the applied piezo-phototronic effect.

**Figure 11:**
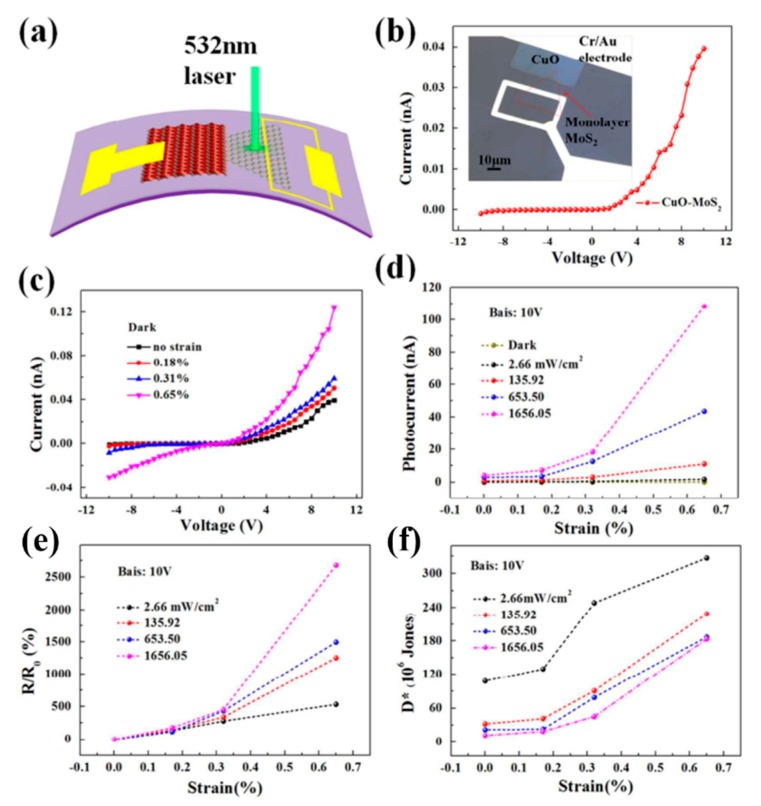
(**a**) The structure diagram of p-CuO/n-MoS_2_ heterojunction PDs on the PET substrate. (**b**) I-V curve of p-CuO/n-MoS_2_ heterojunction without strain in the dark. The inset is the optical image of the PD device. (**c**) I-V curves of the heterojunction with different tensile strains in the dark. (**d**) The strain dependence of the photocurrent. (**e**) The strain dependence of R/R_0_. (**f**) The strain dependence of detectivity with different power densities at a bias of 10 V [125].

Inspired by the enhanced performance caused by the piezo-phototronic effect, a hybrid PD based on heterojunction structure was constructed [72,73]. Zhu and colleagues [72] fabricated broad-band UV/visible/near-infrared self-powered PDs based on p-P3HT and n-CdS. When a 0.67% tensile strain was applied in the [001] direction of CdS contacting with P3HT, the photocurrent of the PDs could be increased to over 330% under UV illumination. This improvement by means of the piezo-phototronic effect could be ascribed to the increased built-in electric field in the interface of the p–n junction, which was more conducive to the separation of photogenerated carriers in CdS and P3HT. It has been reported that the piezoelectric effect that exists in MoS_2_ with an odd number of atomic layers could tune the height of the Schottky barrier in metal-semiconductor contact and/or drive the nanometer device due to its non-central symmetric structure [123,124]. Wang et al. [125] fabricated a flexible photodetector based on a p-CuO film/n-MoS_2_ monolayers heterojunction; the structure diagram and the optical image of PDs are shown in Figure 11a and the inset of Figure 11b, respectively. Figure 11b shows the I-V characteristics of p-CuO/n-MoS_2_ heterojunction had good rectification characteristics in the dark without strain condition. Even at a forward bias voltage of 10 V, it had an ultra-low dark current of 0.039 nA, which was conducive to obtain high-performance optical sensing. Figure 11c exhibits the relationship between current and voltage of the heterojunction with different tensile strains in the dark. The dark current increased from 0.039 nA to 0.12 nA with the increase of tensile strain from 0% to 0.65% at 10 V bias, which resulted from the piezotronic effect of monolayer MoS_2_ (the positive piezopotential caused by tensile strain can modulate the bandgap at the interface of the CuO and MoS_2_ heterojunction). The relation between photocurrent and different tensile strains under different power density of 532 nm laser illumination is shown in Figure 11d; the photocurrent obviously increased with the increased strain due to the piezo-phototronic effect. The strain dependence of R/R_0_ and detectivity of heterojunction PDs with different power densities at a bias of 10 V are displayed in Figure 11e,f, respectively. The photoresponsivity and detectivity of the PDs increased with increasing strain because of the piezo-phototronic effect.

**Figure 12:**
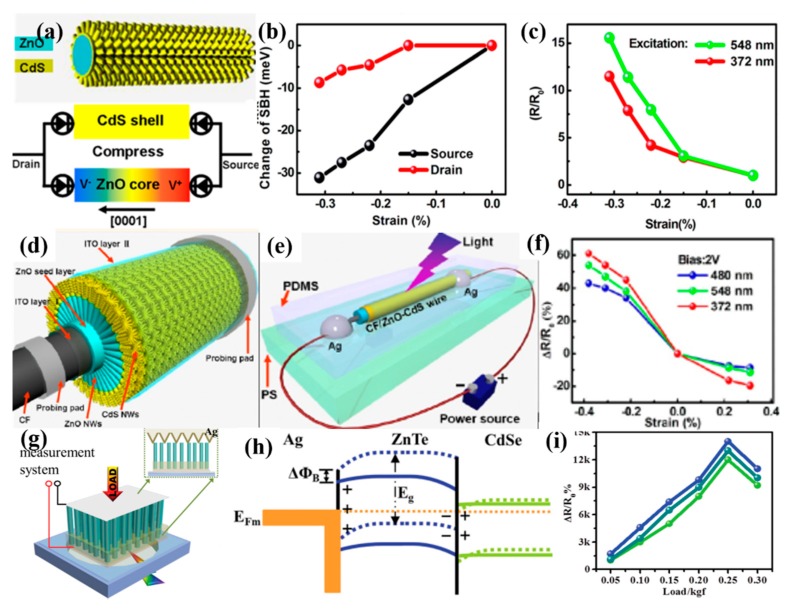
(**a**) Schematic diagram of ZnO/CdS core-shell structure (top), the sandwich model of the PD device, and simulation of the piezopotential distribution in the ZnO nanowire under compressive strain (bottom). (**b**) Relationship between compressive strain and changing Schottky barrier heights. Black curve and red curve represent the SBHs change with strains at source-drain bias of V = 2 and −2 V, respectively. (**c**) The derived change of responsivity in SBH as a function of compressive strains under illumination of green light (548 nm) and UV light (372 nm); R_0_ represents the responsivity of the PD device without compressive strains. (**d**) Schematic representation of CF/ZnO-CdS wire structure. (**e**) Schematic diagram of the PDs based on a single CF/ZnO-CdS wire. (**f**) The strain dependence of ΔR/R_0_ excited by blue light (480 nm), green light (548 nm), and UV light (372 nm); R_0_ is the responsivity without strain, and ΔR was set as the variation of responsivity. (**g**) Schematic diagram of CdSe/ZnTe core/shell nanowire array PDs on PVC with illumination source underneath; the inset is a cross-sectional schematic diagram of the assembled PDs. (**h**) Schematic energy band alignment of the Ag/ZnTe/CdSe structure under compressive load and illumination. (**i**) The strain dependence of ΔR/R_0_ under constant illumination [105,106,114].

A pure semiconductor absorbs photons with an energy larger than the band gap, which limits the wide spectral sensitivity in PD devices. In order to remove the restriction, the core/shell system is adopted for adequate light absorption. The core-shell architecture not only effectively utilizes the inherent material properties of individual components but also provides direct electrical conducting channels for the transport of photocarriers and results in reducing recombination losses; thus, it is a promising choice for improving the performance of photodetectors [118,119,126]. Wang et al. [118] investigated the influence of the piezo-phototronic effect on the photosensitivity of visible and UV PDs based on ZnO/CdS core-shell micro/nanowire, which is shown in Figure 12a. The Schottky barrier heights (SBHs) at the source and drain contacts could be adjusted by the strain induced piezopotential in the ZnO core, and the experimental results in Figure 12b demonstrated that SBHs at the source and drain contacts decreased with the increase of compressive strains. Moreover, the responsivity of ZnO/CdS nanowire-based PDs was improved dramatically by more than ten times with −0.31% compressive strain, as shown in Figure 12c. On this basis, the research group fabricated the branched ZnO/CdS double-shell nanowires array on carbon fiber (CF/ZnO-CdS wire) [117], as illustrated in Figure 12d,e. As shown in Figure 4f, the responsivity of this single CF/ZnO-CdS wire PD was enhanced about 40%–60% under compressive strain of −0.38% and decreased about 8%–20% under a 0.31% tensile strain. In addition, Zhou et al. [126] fabricated broad band PDs based on a type-II CdSe/ZnTe heterojunction core/shell nanowire array using a 100 nm thin Ag layer as top electrode, as illustrated in Figure 12g. When the core/shell nanowires array was bent under the action of external forces, the positive piezopotential on the stretched surface lowered the Schottky barrier height at the Ag-ZnTe junction (Figure 12h), which resulted in the increase of minority carrier diffusion and more carriers flowing through the interface. Combined with the type-II band alignment at the CdSe–ZnTe interfaces and the small lattice mismatch between the CdSe and ZnTe, the responsivity of this PD was enhanced by four orders of magnitude with an applied load of 0.25 kgf under 1.8 V bias, as shown in Figure 12i. Therefore, it is feasible to optimize the performance of PDs based on a multi-function NW array by the piezoelectric photoelectric effect.

## 4. Conclusions

In conclusion, through surface/interface engineering of nanostructured photodetector devices, advanced nanostructured PDs could be constructed by engineering through modulating photon absorption, charge carrier generation, transport, and collection behaviors. Based on the review, it could be well observed that surface/interface engineering of nanostructured PDs is crucially important for affecting PD performance. More importantly, as well illustrated in the paper, through surface or interface engineering such as surface-state passivation for terminating dangling bonds, surface plasmonic resonance for strong scattering and absorption of incident light, interface carrier-trapping/transport modulation, and piezo-phototronic effects for modulating carrier transport behavior in constructing PD devices, the constructed photodetectors’ performance could be considerably improved as supported by reviewing published literature. Even though this brief review paper lists several key factors in the improvement of the constructed photodetector devices, it could be pointed out that several challenges still exist in the way of commercialization of nanostructured photodetectors as shown in the following:

1. Well controlled fabrication of nanostructured materials with uniform morphology and size for constructing designed photodetectors;
2. Well controlled growth of nanostructures with strong absorption of incident photons;
3. Well controlled surface/interface modification for enhancing carrier transportation;
4. Well applied material properties (such as piezoelectric effects) for modulating carrier behavior.

Finally, we believe that advanced nanostructured photodetectors with excellent performance could be designed and fabricated for practical applications through studying the mentioned issues in depth.

## References

[1] Zhang C., Zhu F., Xu H., Liu W., Yang L., Wang Z., Ma J., Kang Z., Liu Y. (2017). Significant improvement of near-UV electroluminescence from ZnO quantum dot LEDs via coupling with carbon nanodot surface plasmons. Nanoscale.

[2] Oener S.Z., van de Groep J., Macco B., Bronsveld P.C.P., Kessels W.M.M., Polman A., Garnett E.C. (2016). Metal–Insulator–Semiconductor Nanowire Network Solar Cells. Nano Lett..

[3] Gonzalez-Pedro V., Juarez-Perez E.J., Arsyad W.S., Barea E.M., Fabregat-Santiago F., Mora-Sero I., Bisquert J. (2014). General Working Principles of CH_3_NH_3_PbX_3_ Perovskite Solar Cells. Nano Lett..

[4] Katoch A., Abideen Z.U., Kim H.W., Kim S.S. (2016). Grain-Size-Tuned Highly H_2_-Selective Chemiresistive Sensors Based on ZnO–SnO_2_ Composite Nanofibers. ACS Appl. Mater. Interfaces.

[5] Huang L., Zhang Z., Li Z., Chen B., Ma X., Dong L., Peng L.M. (2015). Multifunctional Graphene Sensors for Magnetic and Hydrogen Detection. ACS Appl. Mater. Interfaces.

[6] Wu P., Ameen T., Zhang H., Bendersky L.A., Ilatikhameneh H., Klimeck G., Rahman R., Davydov A.V., Appenzeller J. (2019). Complementary Black Phosphorus Tunneling Field-Effect Transistors. ACS Nano.

[7] Pradhan N.R., Rhodes D., Feng S., Xin Y., Memaran S., Moon B.H., Terrones H., Terrones M., Balicas L. (2014). Field-Effect Transistors Based on Few-Layered α-MoTe_2_. ACS Nano.

[8] Dou L., Yang Y., You J., Hong Z., Chang W.H., Li G., Yang Y. (2014). Solution-processed hybrid perovskite photodetectors with high detectivity. Nat. Commun..

[9] Liang H.L., Mei Z.X., Zhang Q.H., Gu L., Liang S., Hou Y.N., Ye D.Q., Gu C.Z., Yu R.C., Du X.L. (2011). Interface engineering of high-Mg-content MgZnO/BeO/Si for p-n heterojunction solar-blind ultraviolet photodetectors. Appl. Phys. Lett..

[10] Liu C.H., Chang Y.C., Norris T.B., Zhong Z. (2014). Graphene photodetectors with ultra-broadband and high responsivity at room temperature. Nat. Nanotechnol..

[11] Xiong D., Deng W., Tian G., Gao Y., Chu X., Yan C., Jin L., Su Y., Yan W., Yang W. (2019). A piezo-phototronic enhanced serrate-structured ZnO-based heterojunction photodetector for optical communication. Nanoscale.

[12] Oh S., Kim C.K., Kim J. (2018). High Responsivity β-Ga_2_O_3_ Metal–Semiconductor–Metal Solar-Blind Photodetectors with Ultraviolet Transparent Graphene Electrodes. ACS Photonics.

[13] Lin R., Zheng W., Zhang D., Zhang Z., Liao Q., Yang L., Huang F. (2018). High-Performance Graphene/β-Ga_2_O_3_ Heterojunction Deep-Ultraviolet Photodetector with Hot-Electron Excited Carrier Multiplication. ACS Appl. Mater. Interfaces.

[14] Zhu H., Shan C.X., Wang L.K., Zheng J., Zhang J.Y., Yao B., Shen D.Z. (2010). Metal−Oxide−Semiconductor-Structured MgZnO Ultraviolet Photodetector with High Internal Gain. J. Phys. Chem. C.

[15] Shalev E., Oksenberg E., Rechav K., Popovitz-Biro R., Joselevich E. (2017). Guided CdSe Nanowires Parallelly Integrated into Fast Visible-Range Photodetectors. ACS Nano.

[16] Wei Q., Chen J., Ding P., Shen B., Yin J., Xu F., Xia Y., Liu Z. (2018). Synthesis of Easily Transferred 2D Layered BiI3 Nanoplates for Flexible Visible-Light Photodetectors. ACS Appl. Mater. Interfaces.

[17] Zhai T., Fang X., Liao M., Xu X., Li L., Liu B., Koide Y., Ma Y., Yao J., Bando Y. (2010). Fabrication of High-Quality In_2_Se_3_ Nanowire Arrays toward High-Performance Visible-Light Photodetectors. ACS Nano.

[18] Mallows J., Planells M., Thakare V., Bhosale R., Ogale S., Robertson N. (2015). p-Type NiO Hybrid Visible Photodetector. ACS Appl. Mater. Interfaces.

[19] Dias S., Kumawat K., Biswas S., Krupanidhi S.B. (2017). Solvothermal Synthesis of Cu_2_SnS_3_ Quantum Dots and Their Application in Near-Infrared Photodetectors. Inorg. Chem..

[20] Yuan S., Shen C., Deng B., Chen X., Guo Q., Ma Y., Abbas A., Liu B., Haiges R., Ott C. (2018). Air-Stable Room-Temperature Mid-Infrared Photodetectors Based on hBN/Black Arsenic Phosphorus/hBN Heterostructures. Nano Lett..

[21] Sarasqueta G., Choudhury K.R., So F. (2010). Effect of Solvent Treatment on Solution-Processed Colloidal PbSe Nanocrystal Infrared Photodetectors. Chem. Mater..

[22] Ackerman M.M., Tang X., Guyot-Sionnest P. (2018). Fast and Sensitive Colloidal Quantum Dot Mid-Wave Infrared Photodetectors. ACS Nano.

[23] Zheng D., Fang H., Long M., Wu F., Wang P., Gong F., Wu X., Ho J.C., Liao L., Hu W. (2018). High-Performance Near-Infrared Photodetectors Based on p-Type SnX (X = S, Se) Nanowires Grown via Chemical Vapor Deposition. ACS Nano.

[24] Sun Z., Liu Z., Li J., Tai G., Lau S.P., Yan F. (2012). Infrared Photodetectors Based on CVD-Grown Graphene and PbS Quantum Dots with Ultrahigh Responsivity. Adv. Mater..

[25] Ma L., Hu W., Zhang Q., Ren P., Zhuang X., Zhou H., Xu J., Li H., Shan Z., Wang X. (2014). Room-Temperature Near-Infrared Photodetectors Based on Single Heterojunction Nanowires. Nano Lett..

[26] Chen X., Mu W., Xu Y., Fu B., Jia Z., Ren F.F., Gu S., Zhang R., Zheng Y., Tao X. (2019). Highly Narrow-Band Polarization-Sensitive Solar-Blind Photodetectors Based on β-Ga_2_O_3_ Single Crystals. ACS Appl. Mater. Interfaces.

[27] Berencén Y., Prucnal S., Liu F., Skorupa I., Hübner R., Rebohle L., Zhou S., Schneider H., Helm M., Skorupa W. (2017). Room-temperature short-wavelength infrared Si photodetector. Sci. Rep..

[28] Song Y.M., Xie Y., Malyarchuk V., Xiao J., Jung I., Choi K.J., Liu Z., Park H., Lu C., Kim R.H. (2013). Digital cameras with designs inspired by the arthropod eye. Nature.

[29] Kim T.I., McCall J.G., Jung Y.H., Huang X., Siuda E.R., Li Y., Song J., Song Y.M., Pao H.A., Kim R.H. (2013). Injectable, Cellular-Scale Optoelectronics with Applications for Wireless Optogenetics. Science.

[30] Kim M.S., Lee G.J., Kim H.M., Song Y.M. (2017). Parametric Optimization of Lateral NIPIN Phototransistors for Flexible Image Sensors. Sensors.

[31] Li J., Li R., Chiang C.H., Zhong Y., Shen H., Song E., Hill M., Won S.M., Yu K.J., Baek J.M. (2020). Ultrathin, High Capacitance Capping Layers for Silicon Electronics with Conductive Interconnects in Flexible, Long-Lived Bioimplants. Adv. Mater. Technol..

[32] Yu K.J., Gao L., Park J.S., Lee Y.R., Corcoran C.J., Nuzzo R.G., Chanda D., Rogers J.A. (2013). Light Trapping in Ultrathin Monocrystalline Silicon Solar Cells. Adv. Energy Mater.

[33] Nam W.I., Yoo Y.J., Song Y.M. (2016). Geometrical shape design of nanophotonic surfaces for thin film solar cells. Opt. Express.

[34] Ouyang W., Teng F., He J.H., Fang X. (2019). Enhancing the Photoelectric Performance of Photodetectors Based on Metal Oxide Semiconductors by Charge-Carrier Engineering. Adv. Funct. Mater..

[35] Mondal S., Dutta K., Dutta S., Jana D., Kelly A.G., De S. (2018). Efficient Flexible White-Light Photodetectors Based on BiFeO_3_ Nanoparticles. ACS Appl. Nano Mater..

[36] Jin Y., Wang J., Sun B., Blakesley J.C., Greenham N.C. (2008). Solution-Processed Ultraviolet Photodetectors Based on Colloidal ZnO Nanoparticles. Nano Lett..

[37] Fang H., Hu W., Wang P., Guo N., Luo W., Zheng D., Gong F., Luo M., Tian H., Zhang X. (2016). Visible Light-Assisted High-Performance Mid-Infrared Photodetectors Based on Single InAs Nanowire. Nano Lett..

[38] Ren D., Azizur-Rahman K.M., Rong Z., Juang B.C., Somasundaram S., Shahili M., Farrell A.C., Williams B.S., Huffaker D.L. (2019). Room-Temperature Midwavelength Infrared InAsSb Nanowire Photodetector Arrays with Al_2_O_3_ Passivation. Nano Lett..

[39] Ahmadi M., Wu T., Hu B. (2017). A Review on Organic–Inorganic Halide Perovskite Photodetectors: Device Engineering and Fundamental Physics. Adv. Mater..

[40] Barone P.W., Baik S., Heller D.A., Strano M.S. (2005). Near-infrared optical sensors based on single-walled carbon nanotubes. Nat. Mater..

[41] Liu H., Yu M., Qin F., Feng W., Hu P. (2018). Two-Dimensional Nonlayered CuInSe_2_ Nanosheets for High-Performance Photodetectors. ACS Appl. Nano Mater..

[42] Lin S.Y., Haider G., Liao Y.M., Chang C.H., Lin W.J., Su C.Y., Liou Y.R., Huang Y.F., Lin H.I., Chung T.C. (2018). Transient and Flexible Photodetectors. ACS Appl. Nano Mater..

[43] Deng Z., Cao D., He J., Lin S., Lindsay S.M., Liu Y. (2012). Solution Synthesis of Ultrathin Single-Crystalline SnS Nanoribbons for Photodetectors via Phase Transition and Surface Processing. ACS Nano.

[44] Hu L., Yan J., Liao M., Xiang H., Gong X., Zhang L., Fang X. (2012). An Optimized Ultraviolet-A Light Photodetector with Wide-Range Photoresponse Based on ZnS/ZnO Biaxial Nanobelt. Adv. Mater..

[45] Soci C., Zhang A., Xiang B., Dayeh S.A., Aplin D.P.R., Park J., Bao X.Y., Lo Y.H., Wang D. (2007). ZnO Nanowire UV Photodetectors with High Internal Gain. Nano Lett..

[46] Guo Z., Zhou L., Tang Y., Li L., Zhang Z., Yang H., Ma H., Nathan A., Zhao D. (2017). Surface/Interface Carrier-Transport Modulation for Constructing Photon-Alternative Ultraviolet Detectors Based on Self-Bending-Assembled ZnO Nanowires. ACS Appl. Mater. Interfaces.

[47] Cao L., Liu X., Guo Z., Zhou L. (2019). Surface/Interface Engineering for Constructing Advanced Nanostructured Light-Emitting Diodes with Improved Performance: A Brief Review. Micromachines.

[48] Many A. (1973). Relation between physical and chemical processes on semiconductor surfaces. CRC Crit. Rev. Solid State Sci..

[49] Nozik A.J., Memming R. (1996). Physical Chemistry of Semiconductor−Liquid Interfaces. J. Phys. Chem..

[50] Han T.H., Tan S., Xue J., Meng L., Lee J.W., Yang Y. (2019). Interface and Defect Engineering for Metal Halide Perovskite Optoelectronic Devices. Adv. Mater..

[51] Hu Z., Wu Z., Han C., He J., Ni Z., Chen W. (2018). Two-dimensional transition metal dichalcogenides: Interface and defect engineering. Chem. Soc. Rev..

[52] Proskuryakov Y.Y., Durose K., Major J.D., Al Turkestani M.K., Barrioz V., Irvine S.J.C., Jones E.W. (2009). Doping levels, trap density of states and the performance of co-doped CdTe(As,Cl) photovoltaic devices. Solar Energy Mater. Solar Cells.

[53] Perlin P., Iota V., Weinstein B.A., Wiśniewski P., Suski T., Eliseev P.G., Osiński M. (1997). Influence of pressure on photoluminescence and electroluminescence in GaN/InGaN/AlGaN quantum wells. Appl. Phys. Lett..

[54] Zhang D., Li C., Han S., Liu X., Tang T., Jin W., Zhou C. (2003). Ultraviolet photodetection properties of indium oxide nanowires. Appl. Phys. A.

[55] Hu L., Yan J., Liao M., Wu L., Fang X. (2011). Ultrahigh External Quantum Efficiency from Thin SnO_2_ Nanowire Ultraviolet Photodetectors. Small.

[56] Chen Q., Ding H., Wu Y., Sui M., Lu W., Wang B., Su W., Cui Z., Chen L. (2013). Passivation of surface states in the ZnO nanowire with thermally evaporated copper phthalocyanine for hybrid photodetectors. Nanoscale.

[57] Cammi D., Ronning C. (2014). Persistent Photoconductivity in ZnO Nanowires in Different Atmospheres. Adv. Condens. Matter Phys..

[58] Pezeshki A., Hosseini Shokouh S.H., Raza S.R.A., Kim J.S., Min S.W., Shackery I., Jun S.C., Im S. (2014). Top and back gate molybdenum disulfide transistors coupled for logic and photo-inverter operation. J. Mater. Chem. C.

[59] Hosseini Shokouh S.H., Pezeshki A., Raza S.R.A., Choi K., Min S.W., Jeon P.J., Lee H.S., Im S. (2014). Molybdenum Disulfide Nanoflake–Zinc Oxide Nanowire Hybrid Photoinverter. ACS Nano.

[60] Hassan M.S., Bera S., Gupta D., Ray S.K., Sapra S. (2019). MoSe_2_–Cu_2_S Vertical p–n Nanoheterostructures for High-Performance Photodetectors. ACS Appl. Mater. Interfaces.

[61] Xiang D., Han C., Hu Z., Lei B., Liu Y., Wang L., Hu W.P., Chen W. (2015). Surface Transfer Doping-Induced, High-Performance Graphene/Silicon Schottky Junction-Based, Self-Powered Photodetector. Small.

[62] Zhou J., Gu Y., Hu Y., Mai W., Yeh P.H., Bao G., Sood A.K., Polla D.L., Wang Z.L. (2009). Gigantic enhancement in response and reset time of ZnO UV nanosensor by utilizing Schottky contact and surface functionalization. Appl. Phys. Lett..

[63] Shen T., Li B., Zheng K., Pullerits T., Cao G., Tian J. (2018). Surface Engineering of Quantum Dots for Remarkably High Detectivity Photodetectors. J. Phys. Chem. Lett..

[64] Fang F., Zhao D.X., Li B.H., Zhang Z.Z., Zhang J.Y., Shen D.Z. (2008). The enhancement of ZnO nanowalls photoconductivity induced by CdS nanoparticle modification. Appl. Phys. Lett..

[65] Garg M., Tak B.R., Rao V.R., Singh R. (2019). Giant UV Photoresponse of GaN-Based Photodetectors by Surface Modification Using Phenol-Functionalized Porphyrin Organic Molecules. ACS Appl. Mater. Interfaces.

[66] Ni S., Guo F., Wang D., Liu G., Xu Z., Kong L., Wang J., Jiao S., Zhang Y., Yu Q. (2018). Effect of MgO Surface Modification on the TiO_2_ Nanowires Electrode for Self-Powered UV Photodetectors. ACS Sustain. Chem. Eng..

[67] Lin J., Zhong J., Zhong S., Li H., Zhang H., Chen W. (2013). Modulating electronic transport properties of MoS_2_ field effect transistor by surface overlayers. Appl. Phys. Lett..

[68] Ai N., Zhou Y., Zheng Y., Chen H., Wang J., Pei J., Cao Y. (2013). Achieving high sensitivity in single organic submicrometer ribbon based photodetector through surface engineering. Org. Electron..

[69] Shen Y., Yan X., Si H., Lin P., Liu Y., Sun Y., Zhang Y. (2016). Improved Photoresponse Performance of Self-Powered ZnO/Spiro-MeOTAD Heterojunction Ultraviolet Photodetector by Piezo-Phototronic Effect. ACS Appl. Mater. Interfaces.

[70] Lu S., Qi J., Liu S., Zhang Z., Wang Z., Lin P., Liao Q., Liang Q., Zhang Y. (2014). Piezotronic Interface Engineering on ZnO/Au-Based Schottky Junction for Enhanced Photoresponse of a Flexible Self-Powered UV Detector. ACS Appl. Mater. Interfaces.

[71] Zhang Z., Liao Q., Yu Y., Wang X., Zhang Y. (2014). Enhanced photoresponse of ZnO nanorods-based self-powered photodetector by piezotronic interface engineering. Nano Energy.

[72] Yu X.X., Yin H., Li H.X., Zhang W., Zhao H., Li C., Zhu M.Q. (2017). Piezo-phototronic effect modulated self-powered UV/visible/near-infrared photodetectors based on CdS:P3HT microwires. Nano Energy.

[73] Peng W., Wang X., Yu R., Dai Y., Zou H., Wang A.C., He Y., Wang Z.L. (2017). Enhanced Performance of a Self-Powered Organic/Inorganic Photodetector by Pyro-Phototronic and Piezo-Phototronic Effects. Adv. Mater..

[74] Liu X., Yang X., Gao G., Yang Z., Liu H., Li Q., Lou Z., Shen G., Liao L., Pan C. (2016). Enhancing Photoresponsivity of Self-Aligned MoS_2_ Field-Effect Transistors by Piezo-Phototronic Effect from GaN Nanowires. ACS Nano.

[75] Lopez-Sanchez O., Lembke D., Kayci M., Radenovic A., Kis A. (2013). Ultrasensitive photodetectors based on monolayer MoS_2_. Nat. Nanotechnol..

[76] Chang Y.H., Zhang W., Zhu Y., Han Y., Pu J., Chang J.K., Hsu W.T., Huang J.K., Hsu C.L., Chiu M.H. (2014). Monolayer MoSe_2_ Grown by Chemical Vapor Deposition for Fast Photodetection. ACS Nano.

[77] Zhang W., Chiu M.H., Chen C.H., Chen W., Li L.J., Wee A.T.S. (2014). Role of Metal Contacts in High-Performance Phototransistors Based on WSe_2_ Monolayers. ACS Nano.

[78] Tan H., Fan Y., Zhou Y., Chen Q., Xu W., Warner J.H. (2016). Ultrathin 2D Photodetectors Utilizing Chemical Vapor Deposition Grown WS_2_ With Graphene Electrodes. ACS Nano.

[79] Thakar K., Mukherjee B., Grover S., Kaushik N., Deshmukh M., Lodha S. (2018). Multilayer ReS_2_ Photodetectors with Gate Tunability for High Responsivity and High-Speed Applications. ACS Appl. Mater. Interfaces.

[80] Tongay S., Zhou J., Ataca C., Liu J., Kang J.S., Matthews T.S., You L., Li J., Grossman J.C., Wu J. (2013). Broad-Range Modulation of Light Emission in Two-Dimensional Semiconductors by Molecular Physisorption Gating. Nano Lett..

[81] Baugher B.W.H., Churchill H.O.H., Yang Y., Jarillo-Herrero P. (2013). Intrinsic Electronic Transport Properties of High-Quality Monolayer and Bilayer MoS_2_. Nano Lett..

[82] Kufer D., Konstantatos G. (2015). Highly Sensitive, Encapsulated MoS_2_ Photodetector with Gate Controllable Gain and Speed. Nano Lett..

[83] Meyer J., Khalandovsky R., Görrn P., Kahn A. (2011). MoO_3_ Films Spin-Coated from a Nanoparticle Suspension for Efficient Hole-Injection in Organic Electronics. Adv. Mater..

[84] Pak S., Jang A.R., Lee J., Hong J., Giraud P., Lee S., Cho Y., An G.H., Lee Y.W., Shin H.S. (2019). Surface functionalization-induced photoresponse characteristics of monolayer MoS_2_ for fast flexible photodetectors. Nanoscale.

[85] Sun Z., Aigouy L., Chen Z. (2016). Plasmonic-enhanced perovskite–graphene hybrid photodetectors. Nanoscale.

[86] Chen Z., Li X., Wang J., Tao L., Long M., Liang S.J., Ang L.K., Shu C., Tsang H.K., Xu J.B. (2017). Synergistic Effects of Plasmonics and Electron Trapping in Graphene Short-Wave Infrared Photodetectors with Ultrahigh Responsivity. ACS Nano.

[87] Luo L.B., Huang X.L., Wang M.Z., Xie C., Wu C.Y., Hu J.G., Wang L., Huang J.A. (2014). The Effect of Plasmonic Nanoparticles on the Optoelectronic Characteristics of CdTe Nanowires. Small.

[88] Li Y., DiStefano J.G., Murthy A.A., Cain J.D., Hanson E.D., Li Q., Castro F.C., Chen X., Dravid V.P. (2017). Superior Plasmonic Photodetectors Based on Au@MoS_2_ Core–Shell Heterostructures. ACS Nano.

[89] Miao J., Hu W., Jing Y., Luo W., Liao L., Pan A., Wu S., Cheng J., Chen X., Lu W. (2015). Surface Plasmon-Enhanced Photodetection in Few Layer MoS_2_ Phototransistors with Au Nanostructure Arrays. Small.

[90] Lin D., Wu H., Zhang W., Li H., Pan W. (2009). Enhanced UV photoresponse from heterostructured Ag–ZnO nanowires. Appl. Phys. Lett..

[91] Arquer F.P.G.d., Beck F.J., Bernechea M., Konstantatos G. (2012). Plasmonic light trapping leads to responsivity increase in colloidal quantum dot photodetectors. Appl. Phys. Lett..

[92] Wang X., Liu K., Chen X., Li B., Jiang M., Zhang Z., Zhao H., Shen D. (2017). Highly Wavelength-Selective Enhancement of Responsivity in Ag Nanoparticle-Modified ZnO UV Photodetector. ACS Appl. Mater. Interfaces.

[93] Hu K., Chen H., Jiang M., Teng F., Zheng L., Fang X. (2016). Broadband Photoresponse Enhancement of a High-Performance t-Se Microtube Photodetector by Plasmonic Metallic Nanoparticles. Adv. Funct. Mater..

[94] Kumar R., Sharma A., Kaur M., Husale S. (2017). Pt-Nanostrip-Enabled Plasmonically Enhanced Broad Spectral Photodetection in Bilayer MoS_2_. Adv. Opt. Mater..

[95] Zhang X., Liu Q., Liu B., Yang W., Li J., Niu P., Jiang X. (2017). Giant UV photoresponse of a GaN nanowire photodetector through effective Pt nanoparticle coupling. J. Mater. Chem. C.

[96] Luo L.B., Zou Y.F., Ge C.W., Zheng K., Wang D.D., Lu R., Zhang T.F., Yu Y.Q., Guo Z.Y. (2016). A Surface Plasmon Enhanced Near-Infrared Nanophotodetector. Adv. Opt. Mater..

[97] Lu J., Xu C., Dai J., Li J., Wang Y., Lin Y., Li P. (2015). Improved UV photoresponse of ZnO nanorod arrays by resonant coupling with surface plasmons of Al nanoparticles. Nanoscale.

[98] Zhang W., Xu J., Ye W., Li Y., Qi Z., Dai J., Wu Z., Chen C., Yin J., Li J. (2015). High-performance AlGaN metal–semiconductor–metal solar-blind ultraviolet photodetectors by localized surface plasmon enhancement. Appl. Phys. Lett..

[99] Knight M.W., Liu L., Wang Y., Brown L., Mukherjee S., King N.S., Everitt H.O., Nordlander P., Halas N.J. (2012). Aluminum Plasmonic Nanoantennas. Nano Lett..

[100] West P.R., Ishii S., Naik G.V., Emani N.K., Shalaev V.M., Boltasseva A. (2010). Searching for better plasmonic materials. Laser Photonics Rev..

[101] Kramer N.J., Schramke K.S., Kortshagen U.R. (2015). Plasmonic Properties of Silicon Nanocrystals Doped with Boron and Phosphorus. Nano Lett..

[102] Zhou S., Ni Z., Ding Y., Sugaya M., Pi X., Nozaki T. (2016). Ligand-Free, Colloidal, and Plasmonic Silicon Nanocrystals Heavily Doped with Boron. ACS Photonics.

[103] Ni Z., Ma L., Du S., Xu Y., Yuan M., Fang H., Wang Z., Xu M., Li D., Yang J. (2017). Plasmonic Silicon Quantum Dots Enabled High-Sensitivity Ultrabroadband Photodetection of Graphene-Based Hybrid Phototransistors. ACS Nano.

[104] Bai S., Jiang W., Li Z., Xiong Y. (2015). Surface and Interface Engineering in Photocatalysis. ChemNanoMat.

[105] Zhu Z., Ju D., Zou Y., Dong Y., Luo L., Zhang T., Shan D., Zeng H. (2017). Boosting Fiber-Shaped Photodetectors via “Soft” Interfaces. ACS Appl. Mater. Interfaces.

[106] Guo F., Yang B., Yuan Y., Xiao Z., Dong Q., Bi Y., Huang J. (2012). A nanocomposite ultraviolet photodetector based on interfacial trap-controlled charge injection. Nat. Nanotechnol..

[107] Ji T., Liu Q., Zou R., Sun Y., Xu K., Sang L., Liao M., Koide Y., Yu L., Hu J. (2016). An Interface Engineered Multicolor Photodetector Based on n-Si (111)/TiO_2_ Nanorod Array Heterojunction. Adv. Funct. Mater..

[108] Song X., Liu X., Yu D., Huo C., Ji J., Li X., Zhang S., Zou Y., Zhu G., Wang Y. (2018). Boosting Two-Dimensional MoS_2_/CsPbBr_3_ Photodetectors via Enhanced Light Absorbance and Interfacial Carrier Separation. ACS Appl. Mater. Interfaces.

[109] Liu Q., Gong M., Cook B., Ewing D., Casper M., Stramel A., Wu J. (2017). Fused Nanojunctions of Electron-Depleted ZnO Nanoparticles for Extraordinary Performance in Ultraviolet Detection. Adv. Mater. Interfaces.

[110] Wang Z.L., Wu W. (2013). Piezotronics and piezo-phototronics: Fundamentals and applications. Natl. Sci. Rev..

[111] Guo Z., Pan H., Li C., Zhang L., Yan S., Zhang W., Yao J., Tang Y., Yang H., Wu Y. (2017). Dynamic carrier transport modulation for constructing advanced devices with improved performance by piezotronic and piezo-phototronic effects: A brief review. Semicond. Sci. Technol..

[112] Yang Q., Liu Y., Pan C., Chen J., Wen X., Wang Z.L. (2013). Largely Enhanced Efficiency in ZnO Nanowire/p-Polymer Hybridized Inorganic/Organic Ultraviolet Light-Emitting Diode by Piezo-Phototronic Effect. Nano Lett..

[113] Wang C., Bao R., Zhao K., Zhang T., Dong L., Pan C. (2015). Enhanced emission intensity of vertical aligned flexible ZnO nanowire/p-polymer hybridized LED array by piezo-phototronic effect. Nano Energy.

[114] Pan C., Niu S., Ding Y., Dong L., Yu R., Liu Y., Zhu G., Wang Z.L. (2012). Enhanced Cu_2_S/CdS Coaxial Nanowire Solar Cells by Piezo-Phototronic Effect. Nano Lett..

[115] Hu G., Guo W., Yu R., Yang X., Zhou R., Pan C., Wang Z.L. (2016). Enhanced performances of flexible ZnO/perovskite solar cells by piezo-phototronic effect. Nano Energy.

[116] Zhu L., Wang L., Xue F., Chen L., Fu J., Feng X., Li T., Wang Z.L. (2017). Piezo-Phototronic Effect Enhanced Flexible Solar Cells Based on n-ZnO/p-SnS Core–Shell Nanowire Array. Adv. Sci..

[117] Zhang F., Niu S., Guo W., Zhu G., Liu Y., Zhang X., Wang Z.L. (2013). Piezo-phototronic Effect Enhanced Visible/UV Photodetector of a Carbon-Fiber/ZnO-CdS Double-Shell Microwire. ACS Nano.

[118] Zhang F., Ding Y., Zhang Y., Zhang X., Wang Z.L. (2012). Piezo-phototronic Effect Enhanced Visible and Ultraviolet Photodetection Using a ZnO–CdS Core–Shell Micro/nanowire. ACS Nano.

[119] Rai S.C., Wang K., Ding Y., Marmon J.K., Bhatt M., Zhang Y., Zhou W., Wang Z.L. (2015). Piezo-phototronic Effect Enhanced UV/Visible Photodetector Based on Fully Wide Band Gap Type-II ZnO/ZnS Core/Shell Nanowire Array. ACS Nano.

[120] Wang Z., Yu R., Wen X., Liu Y., Pan C., Wu W., Wang Z.L. (2014). Optimizing Performance of Silicon-Based p–n Junction Photodetectors by the Piezo-Phototronic Effect. ACS Nano.

[121] Yu R., Pan C., Hu Y., Li L., Liu H., Liu W., Chua S., Chi D., Wang Z.L. (2013). Enhanced performance of GaN nanobelt-based photodetectors by means of piezotronic effects. Nano Res..

[122] Yang Q., Guo X., Wang W., Zhang Y., Xu S., Lien D.H., Wang Z.L. (2010). Enhancing Sensitivity of a Single ZnO Micro-/Nanowire Photodetector by Piezo-phototronic Effect. ACS Nano.

[123] Wu W., Wang L., Li Y., Zhang F., Lin L., Niu S., Chenet D., Zhang X., Hao Y., Heinz T.F. (2014). Piezoelectricity of single-atomic-layer MoS_2_ for energy conversion and piezotronics. Nature.

[124] Zhu H., Wang Y., Xiao J., Liu M., Xiong S., Wong Z.J., Ye Z., Ye Y., Yin X., Zhang X. (2015). Observation of piezoelectricity in free-standing monolayer MoS_2_. Nat. Nanotechnol..

[125] Zhang K., Peng M., Wu W., Guo J., Gao G., Liu Y., Kou J., Wen R., Lei Y., Yu A. (2017). A flexible p-CuO/n-MoS_2_ heterojunction photodetector with enhanced photoresponse by the piezo-phototronic effect. Mater. Horiz..

[126] Rai S.C., Wang K., Chen J., Marmon J.K., Bhatt M., Wozny S., Zhang Y., Zhou W. (2015). Enhanced Broad Band Photodetection through Piezo-Phototronic Effect in CdSe/ZnTe Core/Shell Nanowire Array. Adv. Electron. Mater..

